# Impact of repeat revascularization within 5 years on 10-year mortality after percutaneous or surgical revascularization

**DOI:** 10.1007/s00392-023-02211-6

**Published:** 2023-05-08

**Authors:** Rutao Wang, Mattia Lunardi, Hironori Hara, Chao Gao, Masafumi Ono, Piroze M. Davierwala, David R. Holmes, Friedrich W. Mohr, Nick Curzen, Francesco Burzotta, Robert-Jan van Geuns, Arie Pieter Kappetein, Stuart J. Head, Daniel J. F. M. Thuijs, Ling Tao, Scot Garg, Yoshinobu Onuma, William Wijns, Patrick W. Serruys

**Affiliations:** 1 Department of Cardiology, Xijing Hospital, The Fourth Military Medical University, Xi’an, China; 2 Department of Cardiology, CORRIB Research Center for Advanced Imaging and Core Laboratory, National University of Ireland, Galway (NUIG), University Road, Galway, H91 TK33 Ireland; 3 Department of Cardiology, Radboud University Medical Center, Radboud Institute for Health Sciences, Nijmegen, The Netherlands; 4 The Smart Sensors Laboratory at the Lambe Institute for Translational Medicine and CURAM, University of Galway, Galway, Ireland; 5 Department of Cardiology, University Hospital of Verona, Verona, Italy; 6 Department of Clinical and Experimental Cardiology, Amsterdam UMC, University of Amsterdam, Heart Center, Amsterdam Cardiovascular Sciences, Amsterdam, The Netherlands; 7 Division of Cardiovascular Surgery, Peter Munk Cardiac Centre, Toronto General Hospital, University Health Network, Toronto, Canada; 8 Mayo Clinic, Rochester, MN USA; 9 Department of Cardiac Surgery, Heart Centre Leipzig, Leipzig, Germany; 10 Faculty of Medicine, University of Southampton and Cardiology Department, University Hospital Southampton, Southampton, UK; 11 Fondazione Policlinico Universitario Agostino Gemelli IRCCS, Università Cattolica del Sacro Cuore, Rome, Italy; 12 Department of Cardiothoracic Surgery, Erasmus University Medical Centre, Rotterdam, The Netherlands; 13 East Lancashire Hospitals NHS Trust, Blackburn, Lancashire, UK; 14 NHLI, Imperial College London, London, UK

**Keywords:** All-cause death, Coronary artery bypass grafting, Percutaneous coronary intervention, Repeat revascularization, SYNTAX

## Abstract

**Background:**

The SYNTAX trial demonstrated negative impact of repeat revascularization (RR) on 5-year outcomes following PCI/CABG in patients with three-vessel(3VD) and/or left main coronary artery disease(LMCAD). We aimed to investigate the impact of RR within 5 years, on 10-year mortality in patients with 3VD and/or LMCAD after PCI/CABG.

**Methods:**

The SYNTAXES study evaluated the vital status out to 10 years of patients with 3VD and/or LMCAD. Patients were stratified by RR within 5 years and randomized treatment. The association between RR within 5 years and 10-year mortality was assessed.

**Results:**

A total of 330 out of 1800 patients (18.3%) underwent RR within 5 years. RR occurred more frequently after initial PCI than after initial CABG (25.9% vs. 13.7%, *p < *0.001). Overall, 10-year mortality was comparable between patients undergoing RR and those not (28.2% vs. 26.1%, adjusted HR: 1.17, 95%CI 0.93–1.48, *p = *0.187). In the PCI arm, RR was associated with a trend toward higher 10-year mortality (adjusted HR: 1.29, 95%CI 0.97–1.72, *p = *0.075), while in the CABG arm, the trend was opposite (adjusted HR: 0.74, 95%CI 0.46–1.20, *p = *0.219). Among patients requiring RR, those who underwent PCI as initial revascularization had a higher risk of 10-year mortality compared to initial CABG (33.5% vs. 17.6%, adjusted HR: 2.09, 95%CI 1.21–3.61, *p = *0.008).

**Conclusion:**

In the SYNTAXES study, RR within 5 years had no impact on 10-year all-cause death in the population overall. Among patients requiring any repeat procedures, 10-year mortality was higher after initial treatment with PCI than after CABG. These exploratory findings should be investigated with larger populations in future studies.

**Trial registration:**

URL: https://www.clinicaltrials.gov; SYNTAXES Unique identifier: NCT03417050. URL: https://www.clinicaltrials.gov; SYNTAX Unique identifier: NCT00114972.

**Graphical abstract:**

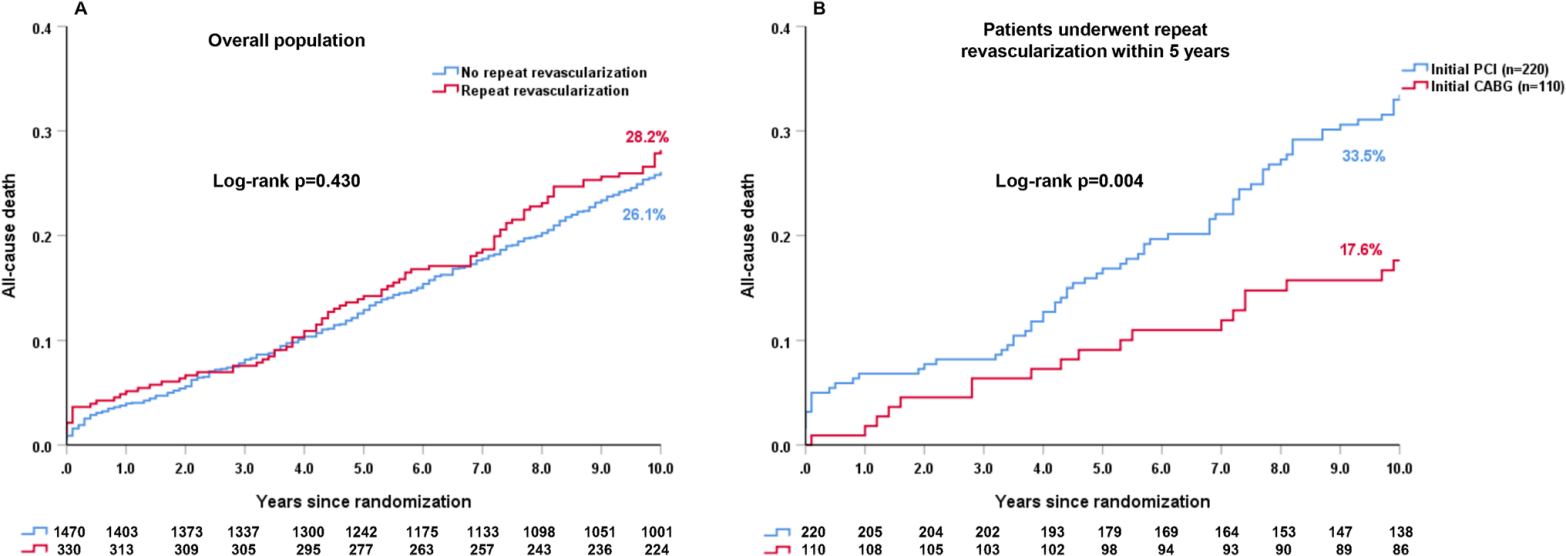

**Supplementary Information:**

The online version contains supplementary material available at 10.1007/s00392-023-02211-6.

## Introduction

The higher rate of additional revascularization required after percutaneous coronary intervention (PCI) compared to coronary artery bypass graft (CABG) has been one of the Achilles' heels of PCI, and this was first addressed by the introduction and routine use of bare metal stents [1–3], with further improvements seen in contemporary practice by their replacement with ever improving drug-eluting stents (DES) [4]. Nevertheless, recent trials comparing PCI to CABG continue to underscore the superiority of CABG in the reduction of this adverse event [4–7].

Repeat revascularization (RR) remains a potential complication in both PCI and CABG patients. Although RR is considered an adverse outcome or failure of the initial treatment, as a strategy it often offers an efficient treatment associated with a reduction in morbidity or mortality [8]. Some authors argue that RR cannot be considered a reliable outcome indicator because of a large number of confounding factors that contribute to the event, ranging from variable indications, the differences in the availability of revascularization targets, to patient preferences [9]. However, the clinical relevance of RR is not negligible, and its negative impact on major adverse events and quality of life has been consistently reported. The SYNTAX trial reported higher rates of the composite endpoint of death, stroke, and myocardial infarction (MI) at 5 years, among patients who were initially randomized to PCI and then underwent secondary revascularization compared to those who did not. A similar, but less impactful, trend was reported among those randomized to CABG [10]. More recently, analyses related to RR in patients with left main (LM) lesions from the EXCEL trial have reaffirmed the association between RR and the risk of 3-year all-cause and cardiovascular mortality after both PCI and CABG [11].

Despite these data, there is currently no evidence as to whether these trends are maintained or amplified long term. Specifically, the impact of RR on all-cause death beyond 5 years has not been fully elucidated. The present study aimed to investigate the impact of RR within 5 years of PCI or CABG on all-cause death among patients with 3VD and/or LMCAD beyond the original 5-year follow-up of SYNTAX trial.

## Methods

### Study population

The SYNTAX study design and the 5-year results have been published previously [5, 12, 13]. The SYNTAX trial completed patient follow-up up to 5 years [13]. The SYNTAXES study was an investigator-driven initiative that extended follow-up using vital status up to 10 years [14]. The extended follow-up was funded by German Heart Research Foundation (GHF; Frankfurt am Main, Germany) and performed in accordance with local regulations of each participating center and complied with the Declaration of Helsinki.

### Endpoints and definitions

The primary endpoint of SYNTAXES study was all-cause death at 10 years. RR was collected within the first 5 years, but no longer recorded after 5 years in SYNTAXES study. The present study is a secondary analysis from the SYNTAXES study assessing the association between RR procedures within 5 years and 10-year all-cause death. In case of patients undergoing more than one RR (regardless of the type), the time-to-first-event was considered for statistical analysis.

The association between the type of RR (PCI or CABG) and 10-year all-cause death was also explored. Patients undergoing RR were categorized into three subgroups according to the type of additional intervention: RR-PCI (patients underwent ≥ 1 RR only by means of PCI), RR-CABG (patients underwent ≥ 1 RR only by means of CABG), and RR-PCI/CABG (patients underwent ≥ 2 RR by means of both PCI and CABG).

Initial PCI and initial CABG refer to the primary revascularization procedure driven by randomization. A staged revascularization procedure was permitted in the SYNTAX trial protocol, provided it was performed ≤ 72 h of the index procedure and during the same hospital stay [12], and these were not considered RR.

### Statistical analysis

Continuous variables are expressed as mean and SD and compared using Student *t* test or Wilcoxon rank sum test as appropriate. Categorical data are reported as counts and percentages, and compared using Chi-square or Fisher’s exact test, when appropriate. Event rates were generated using Kaplan–Meier estimates in time-to-first-event analyses and were compared using log-rank test. Proportional hazard assumption was checked and was not violated in the present study. Therefore, Cox proportional hazard model was applied, and hazard ratio (HR) with 95% confidence interval (CI) was computed. The association of RR with the risk for 10-year all-cause death was evaluated using multivariable Cox models. The covariables in the adjusted Cox models included age, sex, body mass index, prior MI, chronic obstructive pulmonary disease, peripheral vascular disease, medically treated diabetes, chronic kidney disease, congestive heart failure, and anatomic SYNTAX score. All these variables were selected based on prior knowledge of the association of those variables with clinical outcomes [15]. A two-sided *p* value < 0.05 was considered to be statistically significant. All analyses were performed using SPSS Statistics, version 25 (IBM Corp., Armonk, 281 N.Y., USA).

## Results

**Fig. 1:**
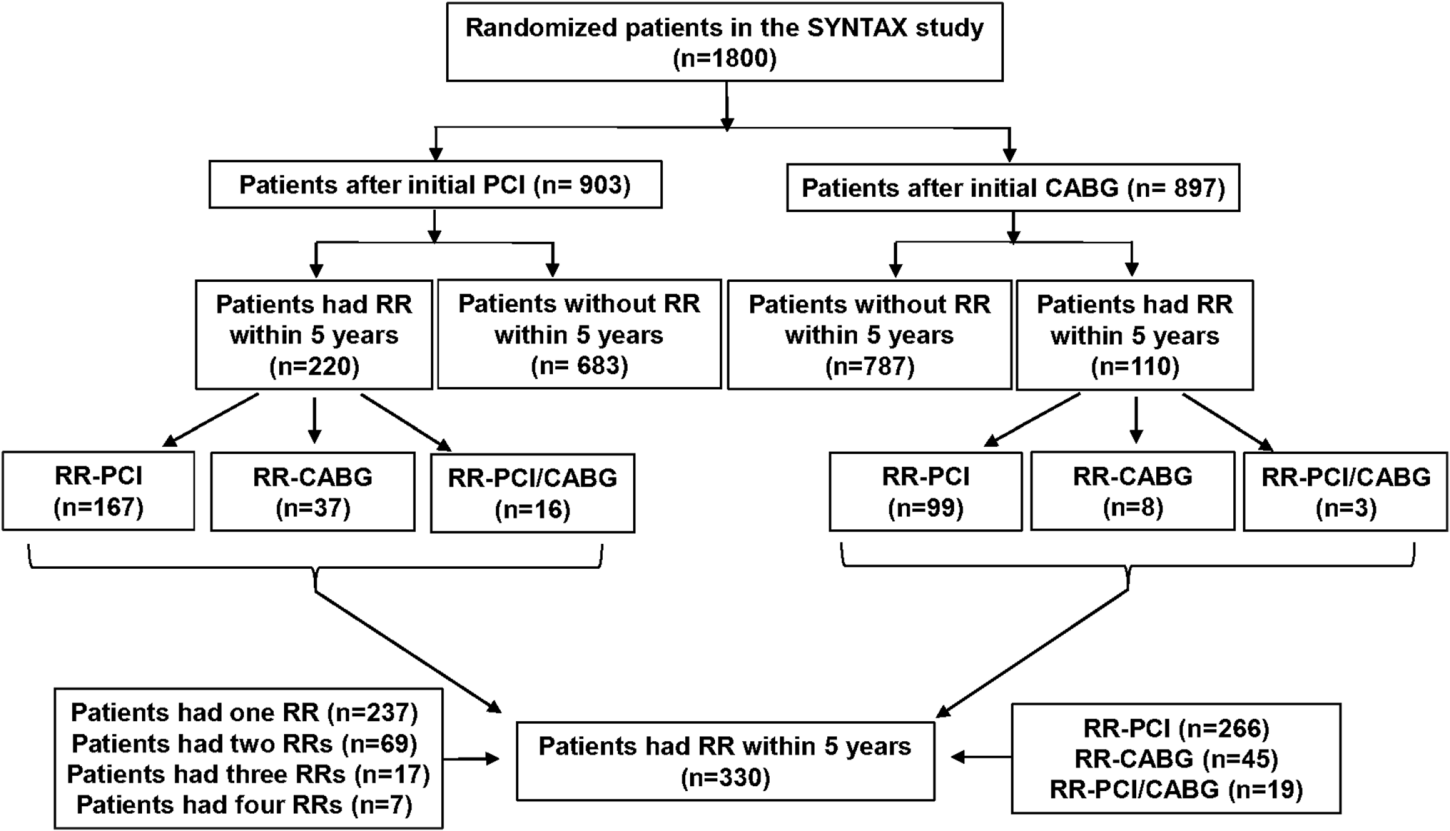
The flowchart of the present study

A total of 330 out of 1800 patients (18.3%) underwent ≥ 1 RR within 5 years of their initial procedures, which amounted to 454 RR procedures (390 additional PCI and 64 additional CABG). The 330 patients undergoing RR were made up of 237, 69, 17, and 7 patients having 1, 2, 3, and 4 RR procedures, respectively (Fig. 1). According to the type of RR, patients were categorized as follows: 266 patients in RR-PCI group, 45 in RR-CABG group, and 19 in RR-PCI/CABG group (Fig. 1).

### Baseline characteristics

Baseline characteristics in patients with vs. without RRs are reported in Tables S1 and S2. In PCI arm, patients undergoing RRs were more likely to be diabetic and had a higher rate of incomplete revascularization; while in CABG group, they had a lower rate of prior MI and a lower logistic EuroSCORE compared to those who did not have RRs (Table S2).

### Impact of RR within 5 years on long-term outcomes

**Fig. 2:**
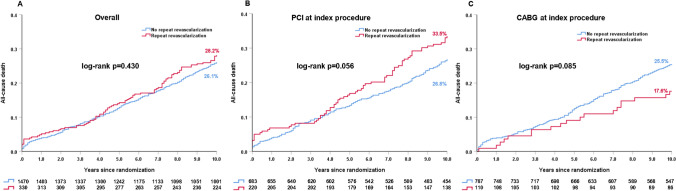
The impact of repeat revascularization within 5 years on 10-year all-cause death. **A** Overall population; **B** PCI arm; **C** CABG arm

In overall population, 10-year all-cause death was comparable between patients who underwent a RR within 5 years and those who did not (28.2% vs. 26.1%, HR: 1.10, 95%CI 0.87–1.38, log-rank *p = *0.430, Fig. 2A). In PCI arm, patients who underwent any RR had a numerically higher rate of 10-year all-cause death compared to those who did not (33.5% vs. 26.8%, HR: 1.30, 95%CI 0.99–1.72, log-rank *p = *0.056, Fig. 2B). By contrast, in CABG population, patients who had any RR had a numerically lower rate of 10-year mortality compared to those who did not (17.6% vs. 25.5%, HR: 0.66, 95%CI 0.41–1.06, log-rank *p = *0.085, Fig. 2C). Of note, the findings were unchanged following adjustment for baseline confounders (Tables 1 and Table 2).

**Table 1:** Association between type of revascularization and 10-year all-cause death in the overall population

	Unadjusted HR	Unadjusted *p*	Adjusted HR	Adjusted *p*
RR (*n = *330)	1.10 (0.87–1.38)	0.430	1.17 (0.93–1.48)	0.187
RR-PCI (*n = *266)	0.98 (0.76–1.27)	0.881	1.04 (0.80–1.35)	0.787
RR-CABG (*n = *45)	2.05 (1.31–3.21)	0.002	1.87 (1.19–2.95)	0.007
RR-PCI/CABG (*n = *19)	0.56 (0.18–1.74)	0.317	0.90 (0.29–2.81)	0.856

*CABG* coronary artery bypass grafting; *PCI* percutaneous coronary intervention; *RR* repeat revascularization

**Table 2:** Association between type of revascularization and 10-year all-cause death in the initial PCI arm and initial CABG arm

	PCI (*n = *903)				CABG (*n = *897)			
	Unadjusted HR	Unadjusted *p*	Adjusted HR	Adjusted *p*	Unadjusted HR	Unadjusted *p*	Adjusted HR	Adjusted *p*
RR (*n = *330)	1.30 (0.99–1.72)	0.057	1.29 (0.97–1.72)	0.075	0.66 (0.41–1.06)	0.087	0.74 (0.46–1.20)	0.219
RR-PCI (*n = *266)	1.27 (0.94–1.72)	0.121	1.28 (0.94–1.74)	0.123	0.51 (0.30–0.88)	0.016	0.56 0.32–0.97)	0.040
RR-CABG (*n = *45)	1.56 (0.93–2.63)	0.094	1.24 (0.72–2.11)	0.438	5.87 (2.41–14.29)	< 0.001	6.94 (2.79–17.30)	< 0.001
RR-PCI/CABG (*n = *19)	0.62 (0.20–1.94)	0.414	0.98 (0.31–3.09)	0.974	–	–	–	–

*CABG* coronary artery bypass grafting; *PCI* percutaneous coronary intervention; *RR* repeat revascularization

### Impact of initial revascularization (PCI or CABG) on long-term outcomes in patients who had RR within 5 years

**Fig. 3:**
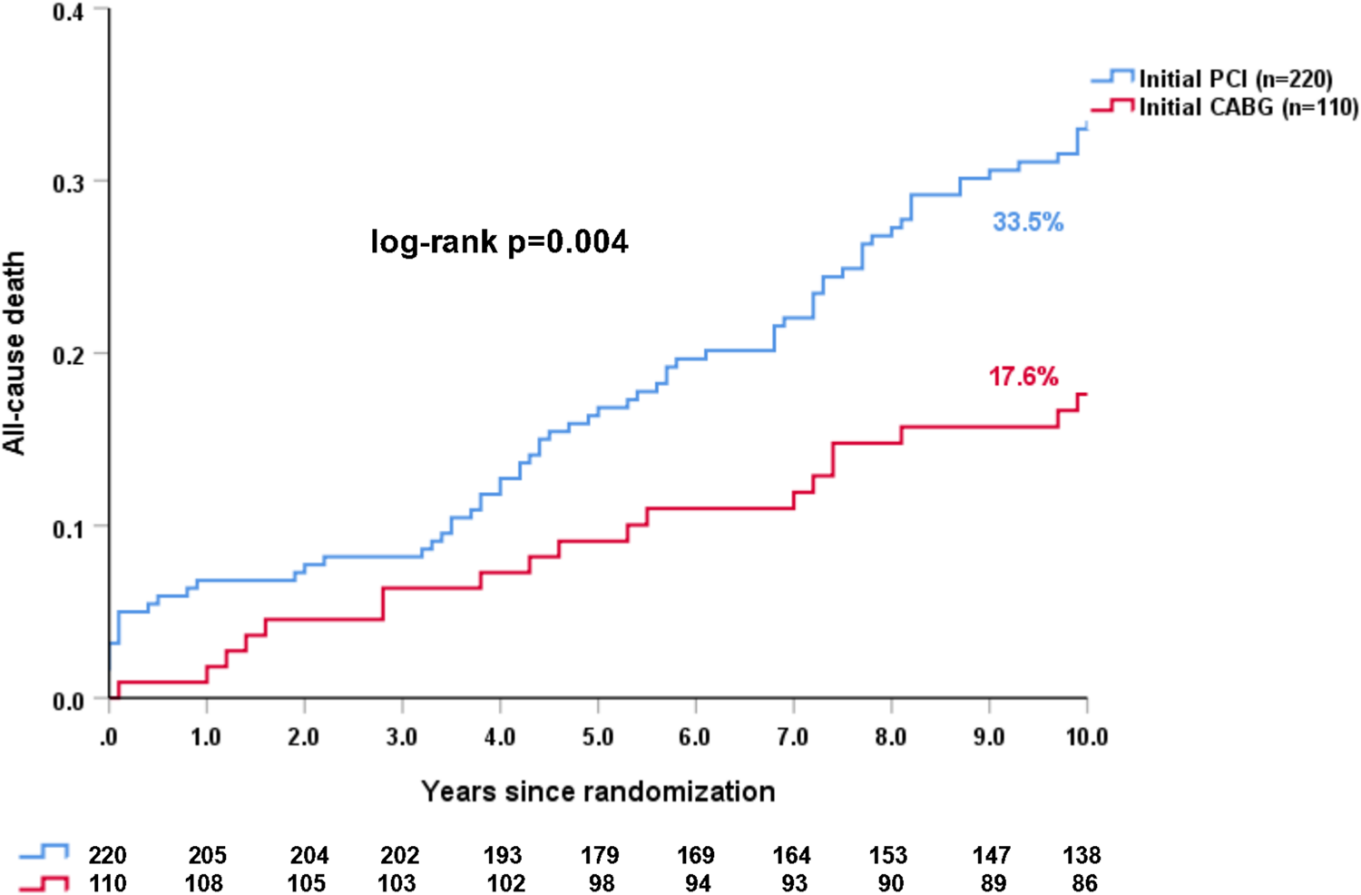
The impact of initial revascularization strategy on 10-year all-cause death in patients who had repeat revascularization within 5 years

**Fig. 4:**
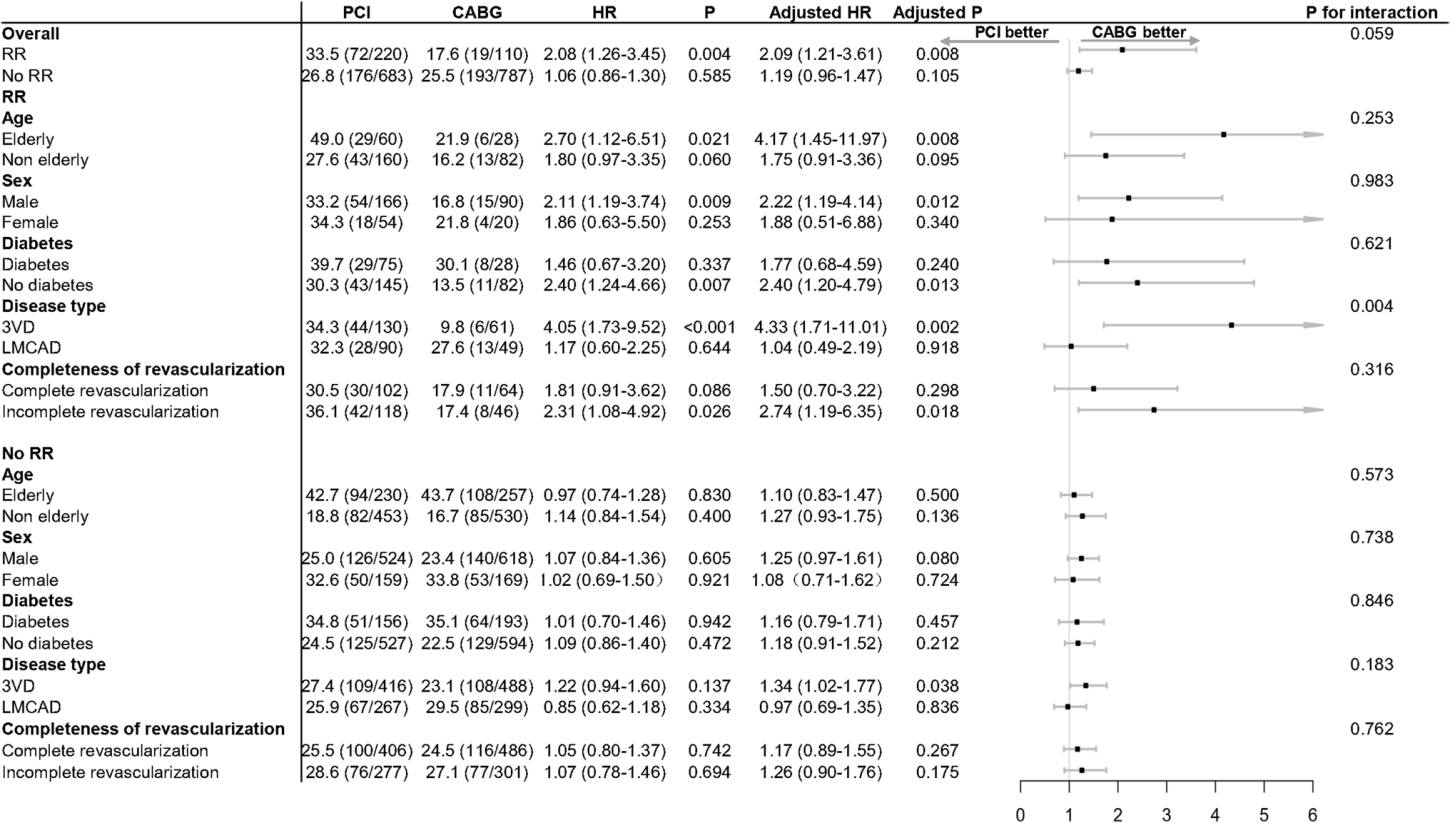
All-cause death at 10 years in the PCI and CABG arms among patients who had repeat revascularization within 5 years or those who did not, stratified by subgroups

The crude rate of 10-year mortality among patients who underwent RR in the first 5 years was higher when the primary mode of revascularization was PCI compared to CABG (33.5% vs. 17.6%, HR: 2.08, 95%CI 1.26–3.45, log-rank *p = *0.004, Fig. 3), with similar findings seen after adjustment for confounding factors (adjusted HR: 2.09, 95%CI 1.21–3.61, *p = *0.008, Fig. 4).

### Risk of 10-year all-cause death in PCI and CABG arms, among patients who had RR within 5 years and those who did not, stratified by subgroups

In terms of long-term survival, there was a trend for a treatment interaction between the initial modality of revascularization and having a RR (p_-interactio*n = *_0.059, Fig. 4). In subgroup analyses among patients who had a RR within 5 years, significant treatment-by-subgroup interactions were observed only in the 3VD/LMCAD subgroups in terms of 10-year mortality (Fig. 4).

### Impact of type of RR within 5 years on 10-year mortality

Independent of initial treatment modality, the risk of 10-year all-cause death was the highest when additional revascularization was performed with CABG only (RR-CABG, *n = *45), followed by PCI only (RR-PCI, *n = *266), and then both PCI and CABG (RR-PCI/CABG, *n = *19) (45.8% vs. 26.1% vs. 15.8%, respectively, log-rank *p = *0.008).

### Multivariable analysis

After adjustment for confounding factors, while RR was not an independent risk factor for 10-year all-cause death, RR with CABG was (adjusted HR: 1.87, 95%CI 1.19–2.95, *p = *0.007, Table 1), whereas RR with PCI was not (Table 1). The high risk of RR with CABG for 10-year all-cause death was mainly contributed by patients whose primary revascularization was with CABG (adjusted HR: 6.94, 95%CI 2.79–17.30, *p < *0.001, Table 2). The association between the type of revascularization and 10-year all-cause death in initial PCI arm and initial CABG arm is shown in Table 2.

### SYNTAX score II 2020 for predicting 10-year all-cause death in patients with RR

**Fig. 5:**
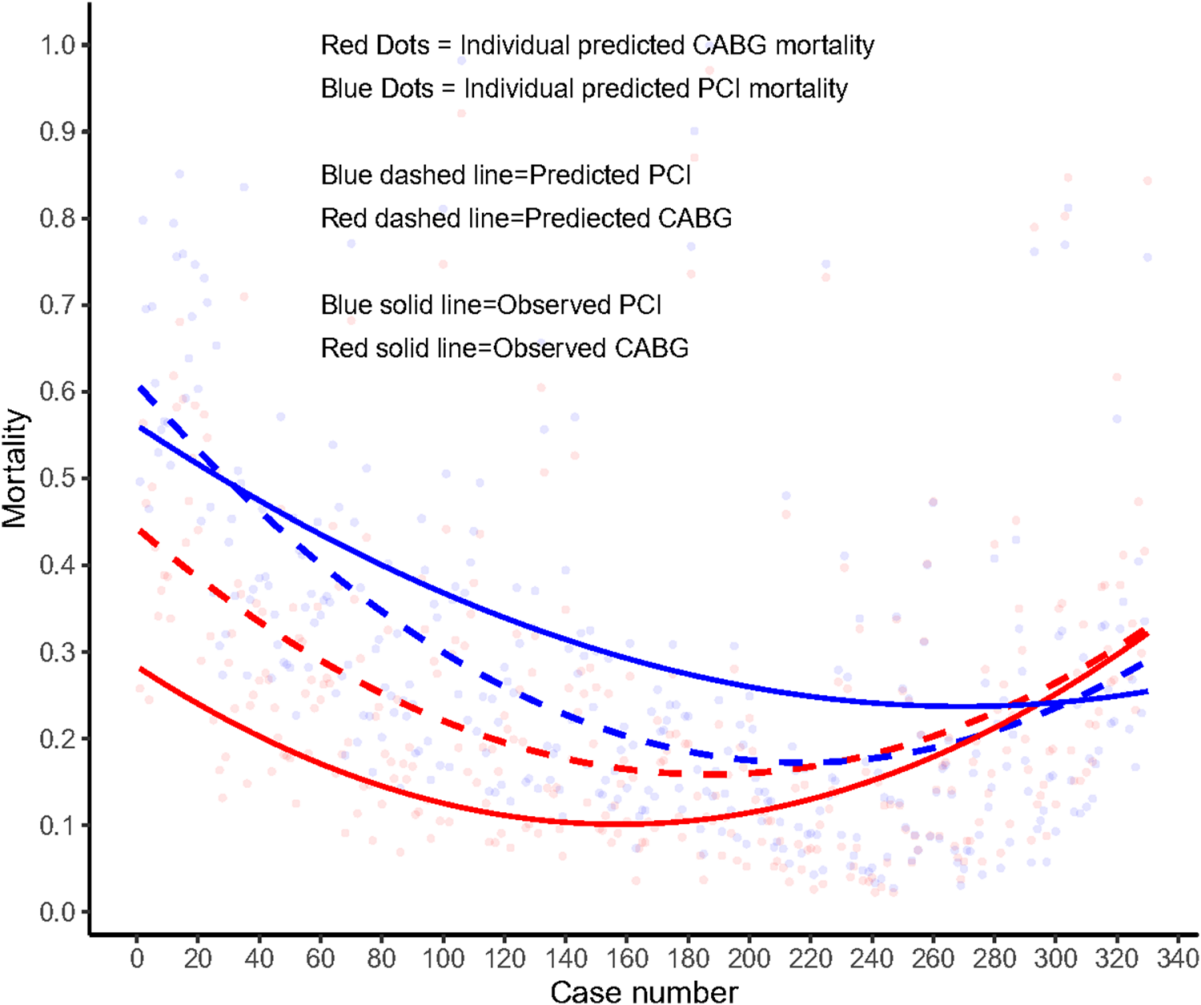
The individual difference between predicted mortality (dashed lines) using the SYNTAX Score II 2020 and the individual observed mortality (solid lines), between initial PCI and initial CABG in patients with repeat revascularization. Blue dashed line represents the predicted mortality after PCI; Red dashed line represents the predicted mortality after CABG; Blue solid line represents the observed mortality after PCI; Red solid line represents the observed mortality after CABG

Figure 5 shows ranked individual differences (*n = *330) in predicted mortality for patients having RR following primary revascularization with PCI (blue dashed line) or CABG (red dashed line). Notably, 229 patients had a predicted mortality which was higher after PCI than CABG; following this, a cross-over point in predicted mortalities (equipoise) was reached, and beyond this, the predicted mortality in the remaining 101 patients was lower following PCI than CABG. The solid line in Fig. 5 depicts, in a spline regression (LOESS) [16], the observed mortality after PCI or CABG. Notably, the solid lines depicting the observed mortalities following either PCI or CABG cross-over the 295th ranked patient suggesting equipoise in vital prognosis after either PCI or CABG for that specific patient.

## Discussion

The present study investigated the association between RR within 5 years of the index PCI or CABG and 10-year all-cause death, among patients with 3VD and/or LMCAD in SYNTAX trial. The main findings are:

1. RR within 5 years did not have impact on 10-year all-cause death.
2. Patients requiring RR had a significantly higher rate of 10-year all-cause death when their primary revascularization was with PCI compared to CABG.
3. Overall, RR was not an independent predictor of 10-year all-cause death. However, RR with CABG was, unlike RR with PCI. The high risk of RR with CABG for 10-year all-cause death was mainly contributed by patients whose primary revascularization was with CABG (RR was a redo CABG).
4. The SYNTAX score II 2020 was able to identify those patients who would benefit the most from either CABG or PCI.

Numerous studies have demonstrated that RR has a negative impact on short- and mid-term outcomes. In patients who underwent elective primary revascularization with isolated CABG, RR with PCI had a poorer prognosis at a median follow-up of 58 months, compared with those who did not [17]. A pooled patient-level analysis from 21 randomized PCI trials demonstrated that target lesion revascularization (TLR) after PCI increased mortality at a median follow-up of 37 months, which was mainly driven by higher rates of MI occurring after TLR [18]. In another patient-level pooled analysis of 1001 patients, TLR was associated with a higher rate of 5-year mortality compared with those without TLR [19]. Similarly in EXCEL trial, the need for a RR increased the risk of 3-year all-cause death after both PCI and CABG (*p*_-interactio*n = *_0.85) [11]. These studies only had medium-term follow-up of 3 to 5 years, the impact of RR on very long-term all-cause death was previously unknown.

In line with the 5-year results of SYNTAX trial [10], we observed a comparable rate of 10-year all-cause death between patients with and without a RR. Whether the impact of RR is related to the mode of primary revascularization is uncertain. In EXCEL trial, among patients having a RR, 3-year all-cause death was numerically higher if the index procedure was with PCI rather than CABG (10.4% vs. 9.1%) [11]. In SYNTAX trial, at 5 years, among patients who underwent RR, those initially randomized to PCI as opposed to CABG had significantly higher rates of the composite of death, MI, or subsequent RR (57.4% vs. 38.4%, *p = *0.003), and a trend for higher mortality (20.2% vs. 13.9%, *p = *0.095) [10]; with the difference in mortality significant by 10 years (Fig. 3). As the value of SYNTAX scores in predicting clinical outcomes [20], we explored SYNTAX score II 2020 for predicting 10-year mortality in patients with RR. Similarly, we demonstrated that the majority of patients (*n = *295) having a RR have a higher rate of 10-year mortality after primary revascularization with PCI than CABG (Fig. 5). SYNTAX score II 2020 clearly identifies those individuals who derive a treatment-specific survival benefit.

There are many possible reasons for these findings. In SYNTAX study, RRs after PCI were mainly target vessel revascularizations, and to a lesser extent treatment of de novo lesions. Disease progression and restenosis appear to be more aggressive after index revascularization with PCI than CABG [10], which may be due to the detrimental effect that coronary stenting has on endothelial function in the distal coronary bed [21, 22]. In addition, bystander enemies, such as diabetes, may contribute to endothelial dysfunction and aggressive disease progression, in particular, in patients who underwent initial PCI, likely influencing their long-term prognosis. As previously reported, indeed, in PCI group, diabetic patients were significantly more frequent among those requiring additional revascularization than those not (34.1% vs. 22.8%, *p < *0.001) [10]. In contrast, bypass grafts to the mid-part of coronary vessels not only removes the vulnerability of proximal lesions, but also potentially offers biological protection and prophylaxis against the development of de novo disease [23].

In addition, initial treatment with CABG offers complete revascularization more frequently than PCI, which may result in a greater protective effect on long-term prognosis [24]. Consistently, PCI patients undergoing RR had a significantly higher incidence of incomplete revascularization than those not requiring RR, whereas such a difference was not observed in the index CABG group (Table S2). This suggests that incomplete revascularization during CABG may not have significant impact on RR risk, probably because most vessels that were not revascularized are either chronically occluded or too small, making their treatment irrelevant. However, the vein graft failure over a 10-year period is high, and might attenuate these benefits. Indeed, multi-arterial CABG is associated with lower mortality [25], which may also partially contribute to the higher rate of 10-year all-cause death after primary revascularization with PCI compared to CABG.

The impact of type of RR on mortality remains uncertain, with previous results inconsistent. A patient-level pooled analysis demonstrated that the type of RR did not affect mortality after TLR of LMCAD [19]. In EXCEL trial, RR using CABG was strongly associated with increased 3-year mortality [11]; in our analysis, after adjustment for confounders, RR-CABG was the only independent predictor of 10-year all-cause death. Of note, the high risk of RR with CABG for 10-year all-cause death was mainly contributed by patients whose primary revascularization was with CABG (Table 2). Furthermore, in our study, the risk of mortality was higher following secondary revascularization with CABG, compared to PCI or both PCI and CABG. Similarly, Locker et al. showed that redo CABG increased 30-day mortality compared to RR with PCI in patients with previous CABG [26]. Given that this result was observed after adjustment, our findings indicate that RR with CABG is a high-risk procedure especially for those with prior CABG, and should, therefore, be considered carefully with the patients. However, considering there were very limited patients who underwent RR with CABG in isolation or following PCI, our findings should be considered as exploratory and hypothesis generating. In addition, RR-CABG patients usually present severe 3VD either not amenable with PCI or where PCI already failed with a large area of myocardium at risk, or yet present non-functioning LIMA-LAD bypass that is a predictor of long-term survival, characteristics that generate biases and preclude definite conclusions. Nevertheless, in patients with previous CABG, the ESC Guideline recommends PCI as the first choice for RR if technically feasible, rather than redo CABG [27]. The recently published ACC/AHA Guideline also recommends that in patients with previous CABG with a patent LIMA to the LAD who need RR, if PCI is feasible, it is reasonable to choose PCI over CABG [28].

At 5 years in SYNTAX trial, patients who underwent RR by PCI had a numerically higher rate of all-cause death compared to those who did not, when their initial revascularization was also by PCI (16.6% vs. 13.2%, *p = *0.26), whereas the opposite was seen after initial CABG (6.3% vs. 12.1%, *p = *0.084) [10]. At 10 years, results were similar, with a trend for increased mortality following primary and secondary revascularization with PCI, and a significantly lower risk when primary CABG was followed by PCI (Table 2). In contrast in EXCEL trial, RR with PCI or CABG were both associated with an increased risk for all-cause death regardless of the initial revascularization approach (PCI or CABG), with no significant interaction observed between the initial revascularization procedure and any type of RR [11]. Ultimately, these inconsistent findings need to be explored in adequately powered clinical studies.

## Limitations

The present study is a post hoc analysis of SYNTAXES trial and may not have adequate statistical power. The main limitation is that RR was no longer recorded after 5 years, we cannot be confident that our findings are reflective of RR in its entirety up to 10 years. Second, the number of patients who underwent RR was limited, especially for those who had repeat CABG or both procedures. Our analysis may lead to the likelihood of spurious findings, all results should be considered exploratory and hypothesis generating. Third, angiographic follow-up was not routinely performed in SYNTAX trial and may underestimate the true rate of RR, especially in patients with silent ischemia. Moreover, not all confounders may have been identified in our multivariable Cox model which assessed the association between RR and all-cause death. Furthermore, the endpoint in SYNTAXES study was all-cause death only; however, death has been considered the most robust and unbiased index for clinical assessment, and is less likely to be affected by ascertainment bias [29]. Finally, the stent-type and peri-interventional therapy used for repeat revascularization were not available. SYNTAX trial enrolled patients with 3VD and/or LMCAD and patients received PCI with first-generation DES; hence, our results should not be extrapolated to general CAD patients in contemporary practice. Further investigations, including latest generation stents and guideline-oriented medical therapy, are warranted.

## Conclusion

In patients with 3VD and/or LMCAD undergoing PCI or CABG, RR within 5 years was not associated with the risk of 10-year all-cause death in the whole population. Among patients requiring any repeat procedures, a higher death rate was observed after primary revascularization with PCI than CABG. The SYNTAX score II 2020 can identify individuals who derive a treatment-specific survival benefit. RR with CABG was associated with an increased risk of 10-year all-cause death especially in patients with previous CABG. These exploratory findings should be investigated in larger populations of patients either pooled retrospectively or enrolled in prospective future studies.

## Supplementary Information

Below is the link to the electronic supplementary material.

Supplementary file1 (PDF 182 kb)

## Data Availability

Data will be made available upon request in adherence to transparency conventions in medical research and through reasonable requests to the corresponding author.
