# Supplementary material for: Impact of repeat revascularization within 5 years on 10-year mortality after percutaneous or surgical revascularization
Source: Clin Res Cardiol. 2023 May 8;112(9):1302–11. doi: 10.1007/s00392-023-02211-6 (PMC10449944; doi:10.1007/s00392-023-02211-6)
Supplement: Supplementary file 1 — Supplementary file1 (PDF 182 kb) [file 392_2023_2211_MOESM1_ESM.pdf]

Supplemental material

1. **Table S1.** Baseline and procedural characteristics according to the need of repeat revascularizations in the overall population.
2. **Table S2.** Baseline and procedural characteristics according to the need of repeat revascularizations after initial PCI and CABG.

Table S1. Baseline and procedural characteristics according to the need of repeat revascularizations in the overall population.

	Overall population (n=1800)		
	**RR (n=330)**	**No RR (n=1470)**	**p**
**Age, yrs**	64.3 ± 9.1	65.3 ± 9.8	0.112
**Female**	22.4 (74)	22.3 (328)	0.965
**Hypertension**	72.1 (238)	65.2 (958)	0.016
**Dyslipidemia**	81.0 (265)	77.2 (1126)	0.133
**Medically treated DM**	31.2 (103)	23.7 (349)	0.005
**Current smoker**	17.6 (58)	20.8 (305)	0.182
**Previous MI**	30.0 (99)	33.1 (486)	0.283
**Peripheral vascular disease**	10.6 (35)	9.7 (142)	0.602
**Previous stroke**	3.3 (11)	4.6 (67)	0.312
**COPD**	8.8 (29)	8.5 (125)	0.867
**Chronic Kidney Disease**	14.5 (48)	18.2 (268)	0.011
**Congestive heart failure**	5.5 (18)	4.5 (65)	0.444
**BMI, kg/m2**	28.2 ± 4.8	28.0 ± 4.7	0.441
**Logistic EuroSCORE**	3.6 ± 2.4	3.8 ± 2.7	0.128
**3VD**	57.9 (191)	61.5 (904)	0.224
**Left main**	42.1 (139)	38.5 (566)	0.224
**SYNTAX Score**	28.9 ± 11.7	28.7 ± 11.4	0.826
**Incomplete revascularization**	49.7 (164)	39.3 (578)	0.001

BMI: body mass index; CABG: coronary artery bypass graft; COPD: chronic obstructive pulmonary disease; DM: diabetes mellitus; MI: myocardial infarction; PCI: percutaneous coronary intervention; RR: repeat revascularization; 3VD: 3-vessel disease.

Table S2. Baseline and procedural characteristics according to the need of repeat revascularizations after initial PCI and CABG.

	PCI (n=903)			CABG (n=897)		
	RR (n=220)	No RR (n=683)	p	RR (n=110)	No RR (n=787)	p
**Age, yrs**	64.8 ± 9.2	65.4±9.8	0.452	63.4 ± 9.0	65.2 ± 9.9	0.069
**Female**	24.5 (54)	23.3 (159)	0.701	18.2 (20)	21.5 (169)	0.428
**Hypertension**	73.6 (162)	67.3 (460)	0.080	69.1 (76)	63.3 (498)	0.234
**Dyslipidemia**	81.6 (177)	77.8 (528)	0.233	80.0 (88)	76.8 (598)	0.449
**Medically treated DM**	34.1 (75)	22.8 (156)	0.001	25.5 (28)	24.5 (193)	0.832
**Current smoker**	15.9 (35)	19.3 (132)	0.256	20.9 (23)	22.2 (173)	0.763
**Previous MI**	32.7 (72)	31.2 (213)	0.669	24.5 (27)	34.7 (273)	0.035
**Peripheral vascular disease**	9.5 (21)	8.9 (61)	0.783	12.7 (14)	10.3 (81)	0.437
**Previous stroke**	3.6 (8)	4.0 (27)	0.821	2.7 (3)	5.1 (40)	0.272
**COPD**	9.1 (20)	7.5 (51)	0.436	8.2 (9)	9.4 (74)	0.679
**Chronic Kidney Disease**	15.5 (34)	19.5 (133)	0.105	12.7 (14)	17.2 (135)	0.301
**Congestive heart failure**	4.6 (10)	3.8 (26)	0.629	7.3 (8)	5.1 (39)	0.335
**BMI, kg/m2**	28.4 ± 4.8	28.0 ± 4.8	0.393	27.9 ±4.8	27.9 ± 4.5	0.921
**Logistic EuroSCORE**	3.7 ± 2.4	3.8 ± 2.7	0.815	3.3 ± 2.3	3.9 ± 2.7	0.032
**3VD**	59.1 (130)	60.9 (416)	0.632	55.5 (61)	62.0 (488)	0.186
**Left main**	40.9 (90)	39.1 (267)	0.632	44.5 (49)	38.0 (299)	0.186
**SYNTAX Score**	29.6 ± 11.4	28.0 ± 11.5	0.081	27.5 ± 12.1	29.3 ± 11.3	0.108
**Incomplete revascularization**	53.6 (118)	40.6 (277)	0.001	41.8 (46)	38.2 (301)	0.471

BMI: body mass index; CABG: coronary artery bypass graft; COPD: chronic obstructive pulmonary disease; DM: diabetes mellitus; MI: myocardial infarction; PCI: percutaneous coronary intervention; RR: repeat revascularization; 3VD: 3-vessel disease
